# Systematic Review and Meta-Analysis of Association of Smokeless Tobacco and of Betel Quid without Tobacco with Incidence of Oral Cancer in South Asia and the Pacific

**DOI:** 10.1371/journal.pone.0113385

**Published:** 2014-11-20

**Authors:** Bhawna Gupta, Newell W. Johnson

**Affiliations:** 1 Population and Social Health Research Programme, Griffith Health Institute, School of Dentistry and Oral Health, Griffith University, Gold Coast, QLD, Australia; 2 Population and Social Health Research Programme, Cancer Research Centre, Griffith Health Institute, Griffith University, Gold Coast, QLD, Australia; University of North Carolina, United States of America

## Abstract

**Aim:**

This systematic review and meta-analysis aimed to critically appraised data from comparable studies leading to quantitative assessment of any independent association between use of oral smokeless tobacco in any form, of betel quid without tobacco and of areca nut with incidence of oral cancer in South Asia and the Pacific.

**Methods:**

Studies (case control and/or cohort) were identified by searching Pub Med, CINAHL and Cochrane databases through June 2013 using the keywords oral cancer: chewing tobacco; smokeless tobacco; betel quid; betel quid without tobacco; areca nut; Asia, the Pacific and the reference lists of retrieved articles. A random effects model was used to compute adjusted summary OR_RE_ for the main effect of these habits along with their corresponding 95% confidence intervals. To quantify the impact of between-study heterogeneity on adjusted main-effect summary OR_RE_, Higgins' H and I_2_ statistics along with their 95% uncertainty intervals were used. Funnel plots and Egger's test were used to evaluate publication bias.

**Results:**

Meta-analysis of fifteen case–control studies (4,553 cases; 8,632 controls) and four cohort studies (15,342) which met our inclusion criteria showed that chewing tobacco is significantly and independently associated with an increased risk of squamous-cell carcinoma of the oral cavity (adjusted main-effect summary for case- control studies OR_RE_ = 7.46; 95% CI = 5.86–9.50, P<0.001), (adjusted main-effect summary for cohort studies RR = 5.48; 95% CI = 2.56–11.71, P<0.001). Furthermore, meta-analysis of fifteen case control studies (4,648 cases; 7,847 controls) has shown betel quid without tobacco to have an independent positive association with oral cancer, with OR = 2.82 (95% CI = 2.35–3.40, P<0.001). This is presumably due to the carcinogenicity of areca nut. There was no significant publication bias.

**Conclusion:**

There is convincing evidence that smokeless (aka chewing) tobacco, often used as a component of betel quid, and betel quid without tobacco, are both strong and independent risk factors for oral cancer in these populations. However, studies with better separation of the types of tobacco and the ways in which it is used, and studies with sufficient power to quantify dose-response relationships are still needed.

## Background

There are more than seventy species of tobacco, where Nicotiana tabacum is the chief commercial crop. This was first introduced into South Asia in the 1600s as a product to be smoked and gradually became popular in many different smokeless forms [1]–[3]. It was not known in Pacific communities before European contact [4] and was introduced to Papua New Guinea by Malay traders [5], [6]. Tobacco in its various forms is frequently shared or exchanged as a way to demonstrate generosity and promote friendship as well as kinship ties in South Asia [2], [7], [8]. Other well-known reasons for chewing tobacco are to seek pharmacologically active stimulants from betel quid or from the tobacco itself to keep chewers awake and/or to relieve stress [8], [9]. Betel quid (BQ) with tobacco, “khaini” (powdered tobacco and slaked lime paste, sometimes with added areca nut) and “gutka” (processed and packaged areca nut with added tobacco) are the most widely used smokeless tobacco (ST) products in the Indian subcontinent (i.e. Pakistan, Bangladesh and India) [10], [11]. BQ, or “paan” as it is known in the Indian language Hindi [12] is one of the four most commonly used psychoactive substances, used by 600 million people around the world [8], [12], [13]. ‘BQ’/‘paan’ is normally defined as ‘a substance, or mixture of substances, placed in the mouth, usually wrapped in betel leaf (derived from the *Piper Betel* vine) with at least one of two basic ingredients: i.e. with/without tobacco and sliced fresh or dried areca nut (*Areca catechu*). The latter is an indispensable ingredient of BQ. The leaves are normally smeared with aqueous lime (calcium hydroxide: derived from shells in coastal areas or from lime deposits inland) in raw or any manufactured or processed form' [14]–[16]. The use of lime lowers the intraoral pH, enhancing the stimulant effect of the nicotine in tobacco [17].

Oral cancer is a disease of multifactorial origin and risk factors vary and operate differently for different population groups. However, the established risk factors are: tobacco in its numerous forms - smoking as well as smokeless/chewing tobacco; areca nut; heavy consumption of alcohol; infection with human papillomavirus; and presence of oral potentially malignant disorders, all of the above frequently having their effects in a background of diets deficient in antioxidant vitamins and minerals [18]–[20].

In this paper, we define oral cancer as any malignant neoplasm arising from the lining mucosae of the lips and mouth (oral cavity), including the anterior two thirds of the tongue. This is defined by the following ICD cancer diagnostic groups: lip and intra-oral sites ICD-10 C00-C06 [21]. The major salivary glands [C07–08], the tonsil [CO9], oropharynx [C10], nasopharynx [C11], pyriform sinus [C12] and hypo-pharynx [C13] are excluded: [C14], ill-defined sites, cannot be realistically considered [22]. Ninety-five per cent of oral cancers are squamous cell carcinomas [23]. Oropharyngeal cancers have been excluded from this meta-analysis in view of their significant association with human papilloma-viruses.

Oral squamous cell carcinoma is more common among countries of South Asia and the Pacific, than in Europe and North America [23], [24] although the spread of these habits amongst the emigrant diaspora is a concern. It is the second most common malignancy among males and sixth among females in the South Asia region as a whole [25]. The highest incidence rates are seen in Papua New Guinea (25.0 per 100,000 per annum), followed by Melanesia as a whole (19.0 per 100,000), Maldives (11.0 per 100,000), Sri Lanka (10.3 per 100,000), Bangladesh (9.4 per 100,000) and India (7.2 per 100,000) [26].

### Aim

The aim of this systematic review with meta-analysis is to critically appraise data from comparable studies, leading to a quantitative summary of the role of ST in its all forms, here designated as ST not otherwise specified (NOS) because of lack of information on the precise nature of the unburned tobaccos product in many published studies, and of betel quid without tobacco, in the aetiology of oral cancer in South Asia and the Pacific.

## Methods

We have followed the Preferred Reporting System for Systematic Reviews (PRISMA) strategy, which specifies systematic selection of articles (as described in detail elsewhere [27], [28] in addition to lessons learned from similar reviews in other fields [29]–[31]. This meta-analysis is based on MOOSE guidelines: Meta-analysis of Observational Studies in Epidemiology [32].

### Search strategy

An extensive computer search of the literature was conducted, including PubMed, CINAHL and Cochrane database. Specific oral cancer sites corresponding to WHO ICD-10 codes C00-C06 were also searched for additional references [33]. Further, reference lists of all full text articles were retrieved and examined in order to obtain additional articles. The following search terms were used: ‘(“Chewing/Smokeless tobacco” OR “Paan” OR “Betel quid” OR “Oral snuff” OR “Khaini” OR “Gutka” OR “Areca nut” for exposure)’ AND ‘(“Oral cancer” OR “Mouth neoplasms” as outcome)’ AND ‘(“India” OR “Taiwan” OR “Bangladesh” OR “Pakistan” OR “Sri Lanka” OR “Nepal” OR “South Asia” OR “Papua New Guinea” OR “the Pacific”)’ as the geographical boundary.

We also performed key-author and reference list searches in order to capture all relevant studies, with no restriction on study type or date of publication. Papers considered were those published in English only.

### Inclusion and exclusion criteria

1) reported original data published in a peer reviewed journal or publicly available with study location, one or both gender specified; *2*) primary outcome was clearly defined as at least some form of malignant neoplasm of lip or oral cavity (ICD10: C00–C06); 3) exposure of interest was smokeless tobacco in any form: (type of ST and whether or not this was combined with BQ was often not given, hence our category of ST NOS) and/or ST with other ingredients unspecified, and/or BQ without tobacco, and/or areca nut alone, areca nut with ST, areca nut mixed with other ingredients unspecified. We found no case-control or cohort studies which explored an association between areca nut alone and oral cancer; 4) sample size was more than 50 cases in a case-control study; 5) provided odds ratios (OR) for case-control studies, or relative risks (RR) for cohort studies, along with their corresponding 95% confidence interval (CI) estimates (or pertinent data for 95% CI computation) as a measure of association between use of ST in its all forms (ST NOS), which therefore includes habitués of BQ plus tobacco, and for BQ without tobacco and its association with oral cancer, adjusted for any of the effects either tobacco smoking, and/or alcohol drinking, age, socioeconomic measures such as education, in the study design or through multivariable logistic regression analysis; 6) results published in English language up to June 2013 and 7) if multiple studies from the same database were published, that with larger sample size and with the highest number of confounders accounted for in the multivariable model were included in our meta-analysis. Because of the absence of much of the above detail in published studies we were forced to create only two categories for meta-analysis, these being ST NOS (which will encompass BQ with tobacco), and BQ without tobacco. We are not aware of any relevant publications since the middle of 2013.

Studies were excluded for the following reasons: 1) studies conducted in the United States of America or Europe; 2) cross-sectional study designs, surveys, case reports, qualitative studies or reviews/meta-analyses; 3) studies with insufficient power (less than five expected exposed cases), appropriate risk estimates and 95% CIs not reported or could not be computed from the available data; 4) unrelated studies, such as pathological and physiological studies on the association between smokeless tobacco and oral cancer; and 5) cohort studies based on mortality of oral cancer and its association with smokeless tobacco in any form [34], [35].

### Data extraction and Quality assessment

The selection process of studies was performed independently by two reviewers (BG and NWJ). The review process encompassed three phases. Consistent with the Cochrane guidelines [36] we chose to err on the safe side during the selection process. Initially, papers were first reviewed based on their titles, followed by study of their abstracts. Those judged to be relevant based on their abstracts were then studied in detail and relevant data gathered. The quality of all publications was assessed based on the STROBE checklist [37].

Details from each article were abstracted by two authors using standardized extraction forms [36]. In case of disagreement, discussion ensued to consensus. We abstracted characteristics relating to the study, type of exposure, health outcome and issues relating to analysis. Where possible, separate effect estimates were obtained for gender for individuals who chew betel quid without tobacco. Estimates for ever-exposure were preferred to crude estimates and where multiple adjusted estimates were available, estimates adjusted for the most potential confounding variables were used.

Crude effect size estimates were derived from the relevant 2×2 table using standard methods, where they were not clearly stated by the authors [38].

### Data synthesis and Meta-analysis

We carried out a meta-analysis under the random effects model which produces results that generalize to a range of populations [39]. For the effect size estimate, standard error of its logarithm was calculated from its reported or estimated confidence interval, assuming that the effect size was log-normally distributed. The logarithms of the effect sizes and their corresponding standard errors formed the data points for random effects meta-analysis [40]. Separate sets of meta-analysis were carried out for case-control studies (ST NOS and for BQ without tobacco use) and cohort studies (ST NOS) in relation to incidence of oral cancer.

### Assessment of Heterogeneity and publication bias

We used a statistical package (Comprehensive meta-analysis version 2) to calculate the summary effect estimate and 95% confidence intervals to test for heterogeneity [41]. For each analysis, within group, heterogeneity was assessed by Cochran's Q statistic (measure of weighted square deviations), with N*-*1 degrees of freedom (where N is the number of studies), result of statistical test based on Q statistic, between studies variance (T2) and ratio of the true heterogeneity to total observed variation (I^2^). Begg's test was used to determine the presence of publication bias. Funnel plots were used to assess publication bias [42], [43].

### Assessment of subgroup analyses

Subgroups were defined by type of study design (case-control or cohort), habit type (ST NOS and BQ without tobacco) and by gender (males and females), study period, cancer site and by whether or not the estimate was reported by the author or derived by us.

## Results

**Figure 1:**
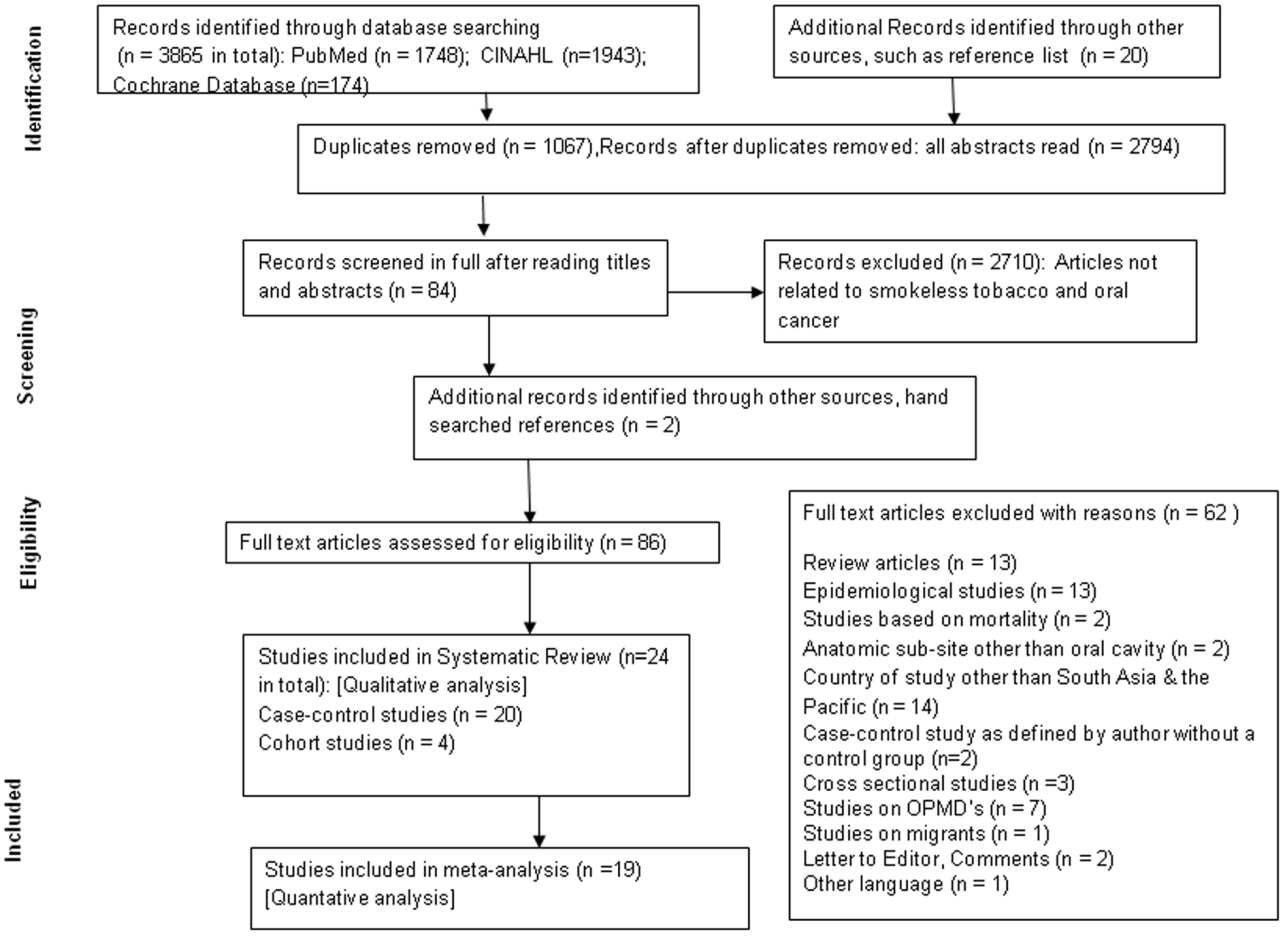
PRISMA strategy for Systematic review and meta-analysis of association of ST and BQ without tobacco with incidence of oral cancer in South Asia and the Pacific.

A total of 3,865 articles were retrieved. After excluding duplicates, 2,794 remained. After studying titles and abstracts, 84 articles were assessed for eligibility. Considering the inclusion and exclusion criteria for selection of manuscripts as described above, twenty four studies were considered for systematic review and nineteen studies were finally able to be included in the meta-analysis (fifteen case-control and four cohort studies as shown in Figure 1).

### Characteristics of the included and excluded studies

All the studies (twenty one case- control and four cohort studies) included for systematic review represent data from South Asia and the Pacific and are presented in Tables 1and 2. Most of these have been conducted in India [15], [16], [44]–[56], one from Pakistan [57] three from Taiwan [58]–[60] and one from Papua New Guinea [61]. Sample sizes ranged from 79 to 2005.

**Table 1:** Study overview (case-control studies included in systematic review).

No	Reference	Region	Time frame of study	Design of research study	Gender	Case Definition (or at risk population)	Controls	Habit	Adjusted factors in analysis
1	Madani A, 2012[45]	India	2005–2006	CCP	M+F	Histopathologically diagnosed incident cases	Relatives, friends, care-takers of cases without cancer	ST NOS, areca nut	Alcohol, non veg diet, education, monthly household income, occupation & other tobacco types
2	Muwonge, 2008[46]	India	1996–2004	CCP	M+F	Histopathologically diagnosed incident cases	Randomly selected, without oral cancer	ST NOS, BQ without tobacco, areca nut/lime+tobacco	Education, religion, smoking & alcohol drinking
3	Thomas SJ, 2007[30]	Papua New Guinea	1985–1987	CCH	M&F	Clinically diagnosed incident cases	Without diagnosis of cancer, plus guardians of inpatients	BQ without tobacco	Age, sex, province, urban or rural residence, educationand income & smoking
4	Zanor, 2003[67]	India	1993–1999	CCH	M	Histopathologically confirmed incident cases	Non tobacco-related cancer patients, histologically confirmed cases	ST NOS, BQ without tobacco	Age, center, education level, alcohol consumption & smoking
5	Balaram P, 2002[48]	India	1996–1999	CCH	M+F	Incident cases by interview & oral examination	Admitted relatives, friends with cancer other than oral cancer, patients from outpatient clinic free from any malignant disease	ST NOS, BQ without tobacco	Age, gender, center, education, smoking & drinking habit
6	Chen PC, 2002[58]	Taiwan	1994–1997	CCH	M&F	Patients diagnosed with SCCC	Not mentioned	ST NOS, BQ without tobacco	Gender, age, smoking, HPV6 & 11
7	Dixit R, 2000[49]	India	1986–1992	CCP	M&F	Cancer registry	Age-stratified, randomly sampled to follow age distribution of cases	ST NOS BQ without tobacco	Religion, educational status, bidi & cigarette smoking
8	Merchant A, 2000[57]	Pakistan	1996–1998	CCH	M&F	Biopsy proven primary cases	Orthopaedics and surgical ward patients without history of any malignancy	ST NOS, BQ without tobacco	Cigarette smoking & alcohol use.
9	Wasnik KS, 1998[50]	India	Not mentioned	CCH	M+F	Clinical, histopathologically and radiologically diagnosed incident cases	One control as non-cancer patient & others having cancer at another site	ST NOS, BQ without tobacco	Smoking and alcohol
10	Lu CT, 1996[59]	Taiwan	1990–1992	CCH	M+F	Patients diagnosed with cancer of oral cavity	Non-cancer cases, living in the same area as the case for at least 5 years and with similar educational background	ST NOS, BQ without tobacco	Alcohol drinking & tobaccosmoking
11	Hirayama, 1996[44]	India, Ceylon, Afghanistan, Thailand, Malaysia, Kasakh SSR and Uzbek SSR	1963–1964	CCH	M+F	Patients diagnosed with cancer of oral cavity	Patients from outpatient clinics of dental department and non cancer patients from same hospital	ST NOS, BQ without tobacco	Not mentioned
12	Ko YC, 1992[60]	Taiwan	1992–1993	CCH	M&F	Histopathologically confirmed	Non-carcinoma patients treated during same period in ophthalmology & physical check-up department	ST NOS, BQ with tobacco	Education & occupation
13	Nandkumar, 1990[15]	India	1982–1984	CCH	M+F	Population based cancer registry	Non-cancer cases	ST NOS, BQ without tobacco	Not mentioned
14	Sankaranarayan, 1990[68]	India	1983–1984	CCH	M+F	Hospital cancer registry	Non-malignant cases & those attending outpatient divisions of teaching hospitals of a medical college	ST NOS, snuff	Age, religion, bidi smoking, snuff & alcohol drinking
15	Sankaranarayan, 1989[51]	India	1983–1984	CCH	M+F	Patients with carcinoma of tongue	Non-malignant cases & those attending outpatient divisions	ST NOS, snuff	Age, religion, bidi smoking, snuff use & alcohol drinking
16	Sankaranarayan, 1989[52]	India	1983–1984	CCH	M+F	Biopsy proven primary cases	Patients attending teaching hospitals with non-malignant diseases	ST NOS, snuff	Age, religion, bidi smoking, snuff use & alcohol drinking
17	Jussawalla DJ, 1971[53]	India	1968	CCP	M&F	Histopathologically confirmed	City residents from registered voters list	ST NOS, BQ without tobacco	Not mentioned
18	Wahi, 1965[54]	India	1950–1962	CCH	M&F	Confirmed cases of oral cancer	Outpatient department for ailments other than H&N cancer	ST NOS	Not mentioned
19	Shanta V, 1963[55]	India	Not mentioned	CCP	M+F	Patients with oral cancer	Non-cancerous patients from exhibitions, fairs & general illness clinics	ST NOS	Not mentioned
20	Shanta V, 1959[56]	India	Not mentioned	CCP	M+F	Patients with cancer	Not mentioned	ST NOS	Not mentioned

Design- CCH  =  case-control with hospital controls, CCP  =  case-control with population controls.

Gender- M+F  =  separate data for males and females, M&F =  gender data combined.

M =  exposure data available only for males.

SCC =  Squamous cell carcinoma.

H&N = Head and Neck.

**Table 2:** Study overview (cohort studies included in systematic review).

No	Reference Type	Region	Time frame of study	Design of research study	Gender	Case Definition (or at risk population)	Years of follow up	Habit
1	Lin JW, 2011[65]	Taiwan	2005–08	HC	M	Patients who visited tertiary referral centre	Not mentioned	BQ without tobacco
2	Jayalekshmi, 2011[62]	India	1990–2005	PC	M	Cancer cases ascertained from Karunagappally cancer registry	End of follow up was date of diagnosis for cancer cases, date of death for all those deceased, or date attaining age of 85	ST NOS
3	Jayalekshmi, 2009[63]	India	1990–2006	PC	F	Cancer cases ascertained from Karunagappally cancer registry	End of follow up was date of diagnosis for cancer cases, date of death for all those deceased, or date attaining age of 86	ST NOS
4	Yen TT, 2008[64]	Taiwan	2005–07	HC	M	Patients who reported to Taichung Veterans General Hospital	Not mentioned	Betel quid without tobacco

Design-HC = Hospital based cohort, PC = Population based cohort.

Gender- M = Males only, F = Females only.

Among cohort studies, two each were from India [62], [63] and Taiwan [64], [65]. There were three cohort studies exclusively for males with two from Taiwan [64], [65]; and one from India [62]. There was only one cohort study from India with data exclusively on females [63]. The sample size of cohort studies ranged from 8,356 to 177,271.

Reasons for exclusion of studies from meta-analysis [66] were: retrospective hospital based review [69], [70]; adjusted OR and statistical analyses were not clearly stated or it was not possible to compute the effect estimate from the information given [54], [71]–[73]; and the sample size of a case-control study was less than 50 [58]. Further cohort studies which reported hazard ratio as an expression of mortality rate from oral cancer were also excluded [34], [35], [74]. For further information please refer to Table S2.

For studies in which effect estimates in terms of OR for case- control and RR for cohort studies were not available [48], [50], [53], [67], [68] these were calculated by constructing two by two tables and using the software MedCalc available at http://www.medcalc.org/calc/odds_ratio.php. Where two or more studies were published from the same database, that with the largest sample size and with the highest number of confounders in the multivariate model was used [16], [51], [52].

### Reported primary outcome: Smokeless tobacco (NOS) and incidence of oral cancer: case-control studies

**Figure 2:**
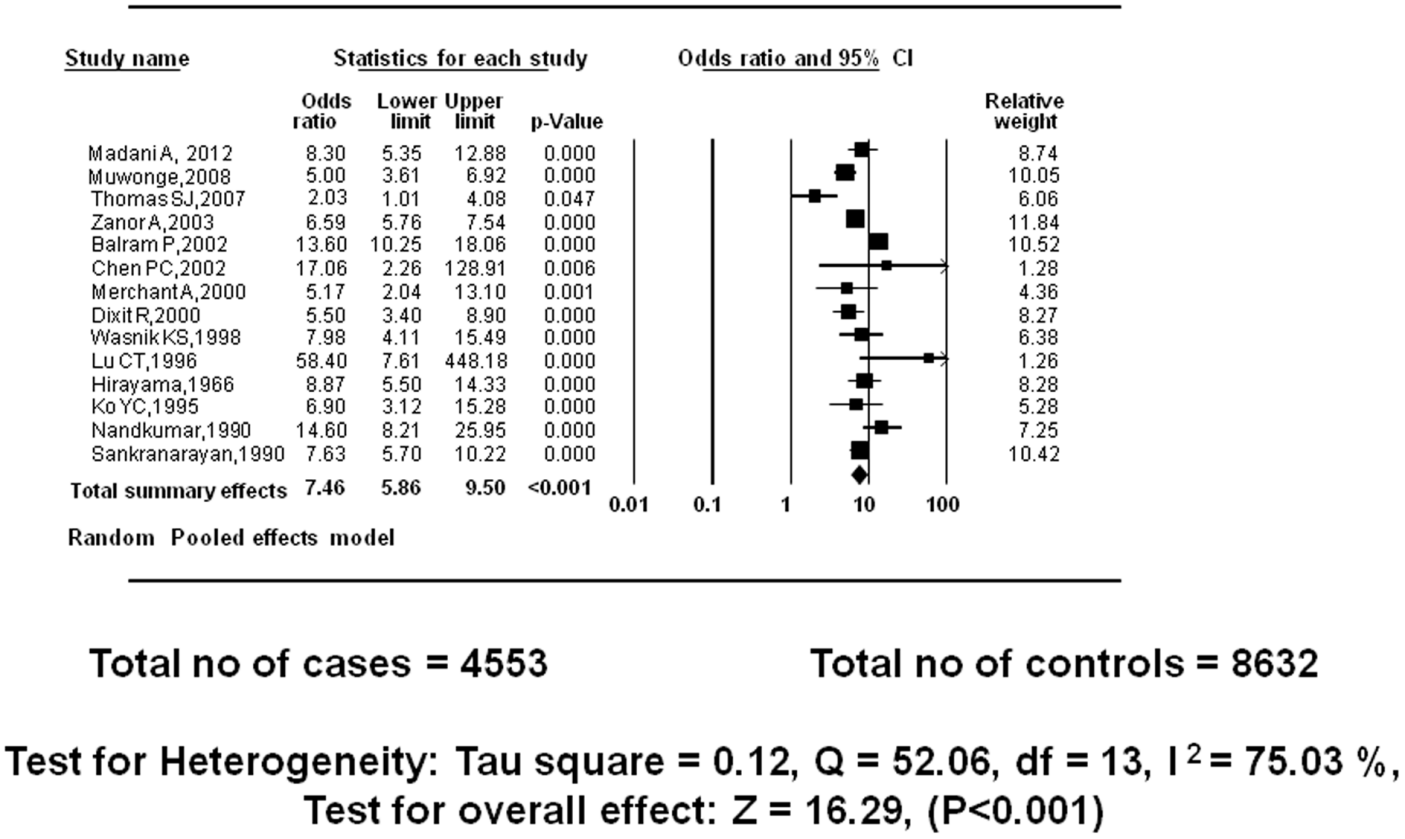
Meta-analysis of case-control studies: ST NOS use and incidence of oral cancer.

Fourteen studies with total sample size of 4,553 cases and 8,632 controls were included in meta-analysis. Under the random effects model, the overall estimate for males and females combined together was OR_RE_ = 7.46 (95% CI = 5.86–9.50, P<0.001) which indicates a very strong association [30]. The test for heterogeneity produced Tau square of 0.12, Q = 52.06, I^2^ = 75.03%, test for overall effect z = 16.29 (Figure 2).

### Reported primary outcome: Betel quid without tobacco and incidence of oral cancer: case-control studies

**Figure 3:**
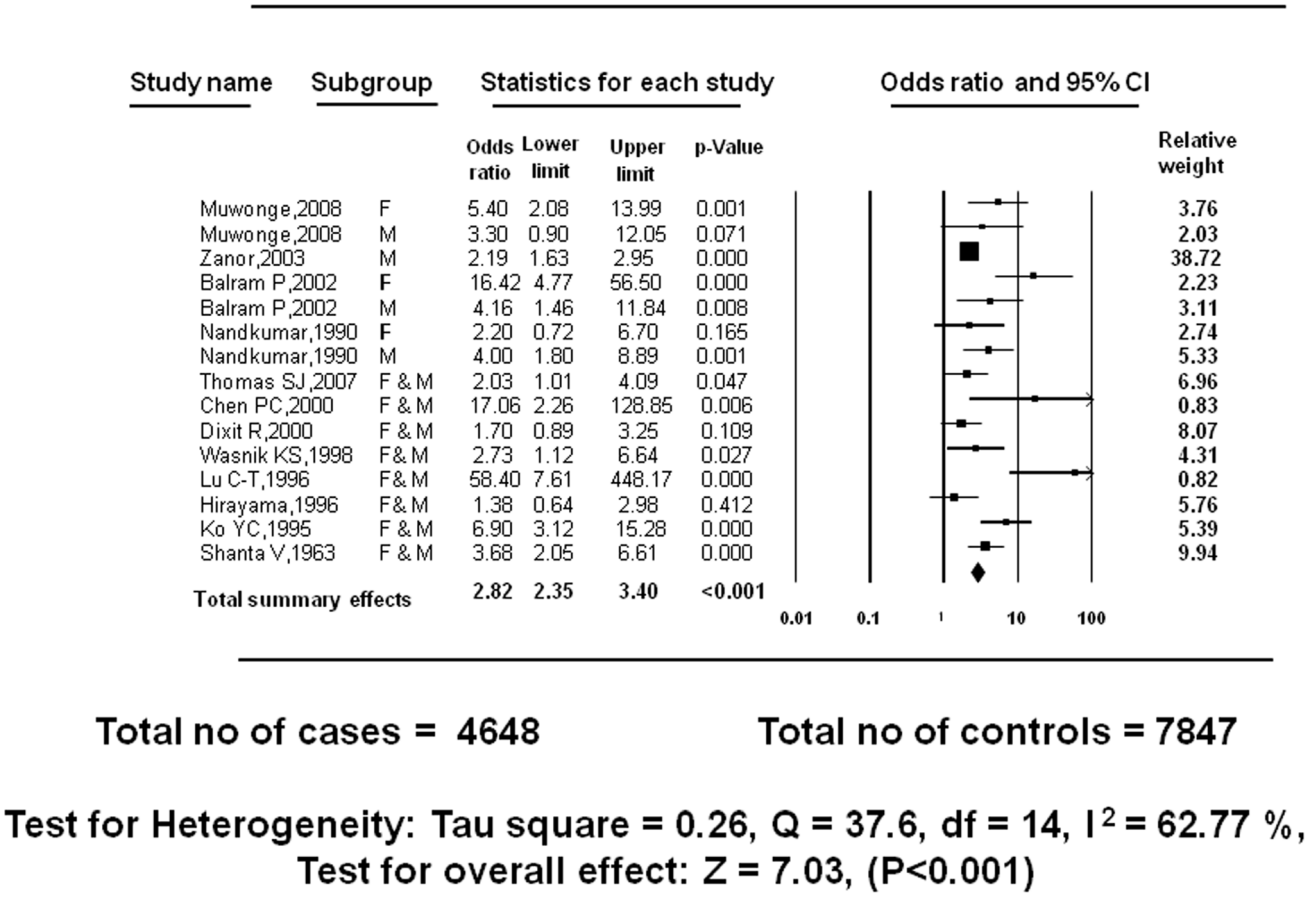
Meta-analysis of case-control studies: BQ without tobacco use and incidence of oral cancer.

Fifteen case-control studies with total sample size of 4,648 cases and 7,847 controls, when stratified on the basis of gender, demonstrate a positive relationship between betel quid without tobacco and incidence of oral cancer. Under the random effects model, the total summary effects computed were OR_RE_ = 2.82 (95% CI = 2.35–3.40, P<0.001). The test for heterogeneity described Tau square = 0.26, Q = 37.6, df = 14, I^2^ = 62.77%, test for overall effect z = 7.03. This I^2^ value is indicative of substantial heterogeneity among the studies (Figure 3).

However, where results when stratified by gender one study reported little or no association between betel quid without tobacco and incidence of oral cancer for females [15], one study for males [46] and one for females and males combined [49]. On the other extreme, a study reported odds of exposure of females using betel quid without tobacco and their related incidence of oral cancer to be sixteen times higher. However, the reported confidence intervals in this study, 4.77–56.50, were too wide to support the precision of evidence in the study, which may be due to small sample size/relative weight and insufficient power [75] (Figure 3).

### Reported primary outcome Smokeless tobacco (NOS) and incidence of oral cancer: cohort studies

**Figure 4:**
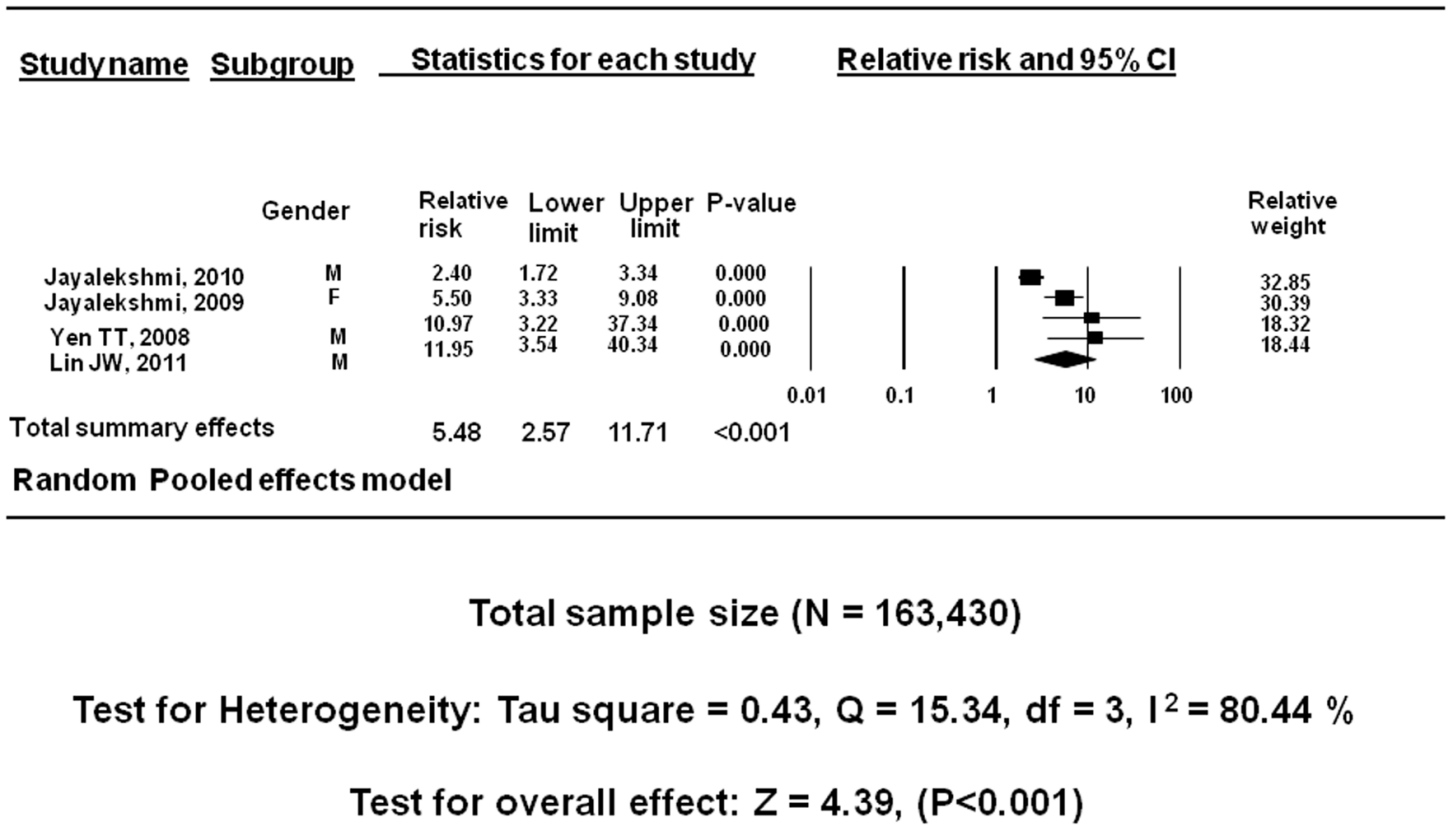
Meta-analysis of cohort studies: ST NOS use and incidence of oral cancer.

Four cohort studies with a total sample size of 163,430 showed a positive relationship between ST NOS and incidence of oral cancer. Under the random effects model, the overall estimate for males and females combined together was RR = 5.48 (95% CI = 2.57–11.71, P<0.001). The test for heterogeneity described Tau square  = 0.43 indicating homogeneous results, Q = 15.341, I^2^ = 80.445, test for overall effect z = 4.395. This I^2^ value is suggestive of considerable heterogeneity (Figure 4).

### Common intra-oral sub-sites for cancer

Most studies reported intra-oral sub-site for cancer, some did not [58], [61], [71]. Studies from India report buccal mucosa as the most common sub-site [15], [16], [44], [53], followed by tongue [15], the least frequent site being lip unspecified. Similarly, buccal mucosa and tongue were the most common sites of oral cancer observed in Taiwan in association with these habits [59], [76] (Table 3).

**Table 3:** Cancer site.

Study number																						
CANCER SITE	1[77]	2[46]	3[78]	4[63]	5[64]	6[67]	7[75]	8[57]	9[49]	10[44]	11[50]	12[76]	13[15]	14[16]	15[51]	16[52]	17[53]	18[54]	19[55]	20[56]	21[59]	22[65]
Oral cavity					•		•										•	•			•	•
Buccal mucosa		√						√	√	√	√			√			√		√	√		
Labial mucosa														√								
Floor of mouth	√	√	√	√		√		√				√	√		√		√			√		
Alveolus													√				√					
Gum	√	√	√	√				√	√			√				√		√		√		
Palate	√	√						√	√								√	√		√		
Lip	√	√				√			√			√	√				√	√	√			
Pyriform sinus									√													
Tongue	√					√		√	√			√	√		√							
Unspecified parts of mouth	√											√										

It is important to note here that the intra oral sites are not listed according to WHO ICD-10-C00-C06, but according to what authors have reported.

-•These studies did not report sub-site, using descriptors such as oral cancer/cancer of oral cavity.

-Floor of mouth followed by lip, tongue and buccal mucosa were the most common sites as determined from this systematic review.

### Sensitivity analyses

To explore the reasons for the observed heterogeneity, sensitivity analyses were performed by grouping studies that showed more similar characteristics, such as similar cases according to ICD-10 codes, cases restricted to exposure to betel quid without tobacco, those that presented disaggregated data by sex, or those that were adjusted for a core of variables, such as age, sex, cigarette smoking, and alcohol consumption. Finally, we investigated the effect of the poor-quality studies on the overall effect size by performing a sensitivity analysis on the results by 2 subgroups, which were based on individual scores above or below the median. For each estimate included, the value of Q^2^ is calculated by *w* (*x* - *x*–)^ 2^, where *w* is the inverse-variance weight, *x* is the logarithm of the effect size and *x*– its mean. Q^2^ is the contribution of the estimate to the heterogeneity chi-squared statistic. Where there is significant (P<0.05) heterogeneity of estimates, sensitivity to potentially outlying estimates is tested by removing that with the largest Q^2^ value and rerunning the analyses. This process was continued until there was no longer significant heterogeneity [77].

### Publication bias

**Figure 5:**
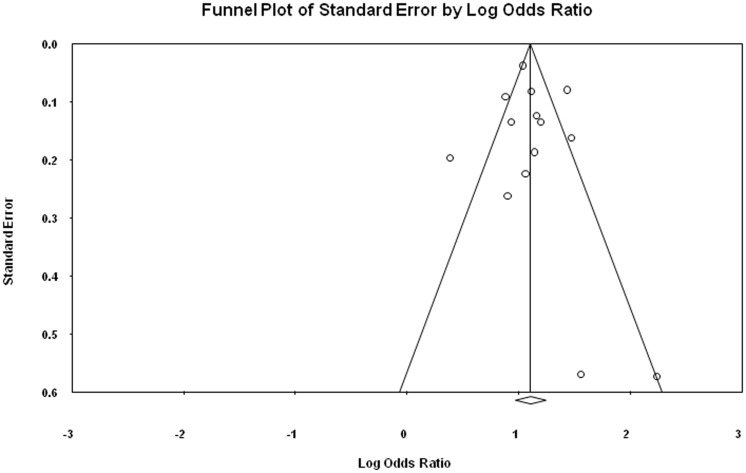
Publication bias for case-control studies illustrating the relationship between ST NOS and incidence of oral cancer.

**Figure 6:**
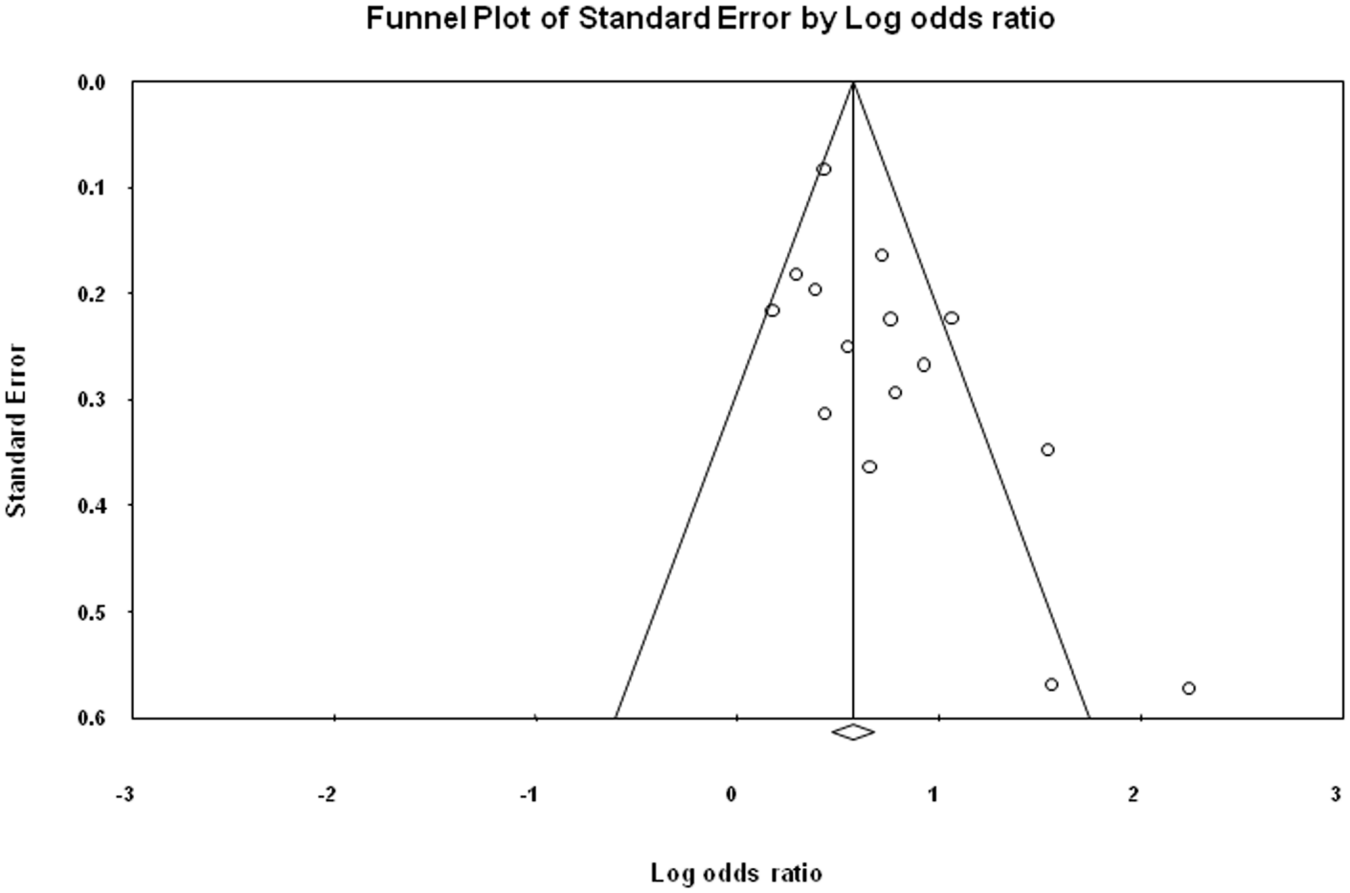
Publication bias for case-control studies illustrating the relationship between BQ without tobacco and incidence of oral cancer.

**Figure 7:**
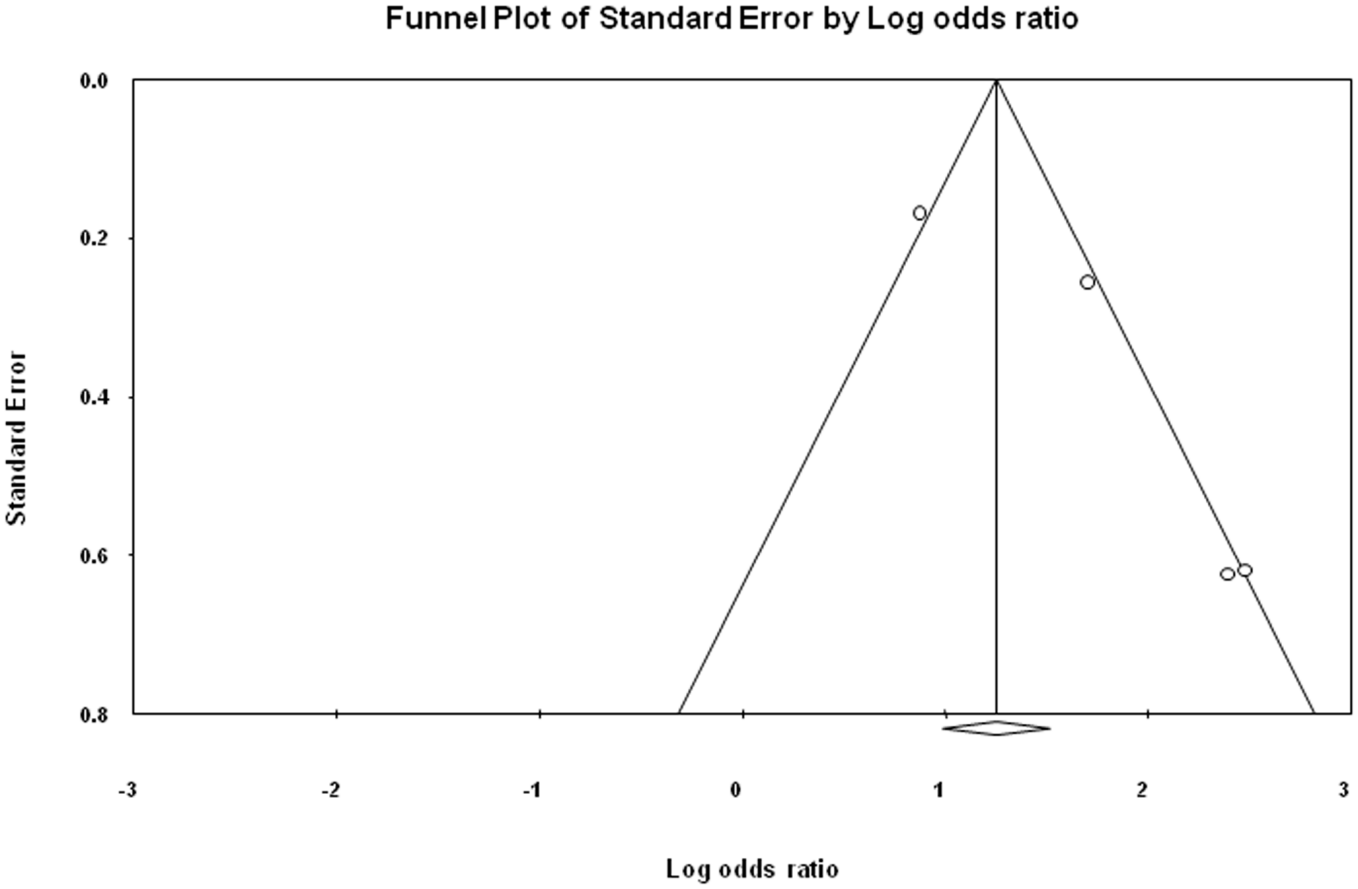
Publication bias for cohort studies illustrating the relationship ST NOS and incidence of oral cancer.

The symmetrical funnel plot for ST NOS, BQ without tobacco and incidence of oral cancer from included case- control and cohort studies indicates that there is no publication bias in our meta-analysis. The publication bias is illustrated in Figure 5, 6 and 7.

Egger regression procedures were further used to test for bias [78], [79]. Egger's regression intercept was 0.49, standard error  = 0.91, 95% confidence interval (CI) were 1.51–2.49, t = 0.53, df = 12 and P-value = 0.60 (Figure 5). Egger's regression intercept was 2.20, standard error = 0.90, 95% CI were 0.26–4.15, t = 2.45, df  = 13 and P-value = 0.03 (Figure 6). Egger's regression intercept was 3.62, standard error  = 1.286, 95% CI were 1.19–9.16, t = 2.82, df = 2 and P-value = 0.05 (Figure 7).

## Discussion

After reviewing all the included observational studies from India, Pakistan, Taiwan and Papua New Guinea exploring the relationship between oral cancer and ST NOS as well as BQ without tobacco, this meta-analysis supports the view that ST NOS and BQ without tobacco are associated with increased risk for oral cancer.

An association between BQ chewing and oral cancer was first identified in 1933 based on a study of 100 oral cancer patients in India [80]. This was later supported by many other studies from Malaysia, Taiwan and the Pacific [54], [81]–[84]. Studies mainly from South Asia have reported the risk of oral cancer and the use of oral tobacco in various forms including “paan” with and without tobacco [15,34,44 51,53, 55,56,71,73, 85–87]. These studies have shown that the tobacco chewing habit increases the risk of oral cancer by seven fold as compared to non- chewers. However, some studies have reported relatively non-significant associations between chewing BQ without tobacco and incidence of oral cancer [15], [46], [49].

The magnitude of risk of oral cancer associated with chewing BQ without tobacco was much higher in Taiwan (mRR, 10.98) than in the Indian subcontinent (mRR, 2.56). This difference may be due to a larger number of quids consumed per day in Taiwan, but also due to region-specific variations in the preparation of betel quid, specifically in the type of areca nut chewed (e.g., preparation, ripeness) as well as the type of slaked lime added. A case-control study from Thailand reported that, among all components of the betel quid, the presence in the quid of red slaked lime had the strongest effect on the risk of oral cancer (OR,10.67; 95% CI = 2.27–50.08) [88].

In Taiwan, generally males who chewed BQ without tobacco were 24 times at a greater risk of developing oral cancer than those who did not chew BQ without tobacco [12], [60], [89]–[91]. In addition, almost all (88%) BQ chewers were smokers, consistent with findings of previous studies conducted in Taiwan [65], [76], [92].

In the Pacific, betel quid is usually consumed without ST, e.g. in Melanesia, whereas parts of the Federated States of Micronesia and in Cambodia, tobacco is usually added [7] with smoking also a common habit.

A linear dose response relationship was observed between number of tobacco quids chewed per day and the risk of oral cancer [15], [44], [49], [50]. This risk increases by nearly thirteen times with increase in duration from 30 to 40 years of chewing tobacco among both sexes. The trend observed is not linear for both the sides of mouth [15], [16], [49]. A study in Taiwan demonstrated that retaining and subsequently swallowing betel quid juice (saliva extract of betel quid produced by chewing) and including unripened betel fruit in the quid both seemed to enhance the risks of contracting oral cancer by eleven times [76]. Several other studies reveal a dose: response relationship between chewing tobacco and oral cancer [35], [47], [62], [63], [67].

A recently published meta-analysis also explores the relationship between betel quid chewing and risk of oral and oropharyngeal cancers [93]. Our results are consistent with this paper in several ways, for example: betel quid without added tobacco in addition to smokeless tobacco in its all forms causes cancer of the oral cavity in humans; there is a clear demonstration of increasing risk of oral cancer with increasing duration of ST NOS and of BQ without tobacco, strengthening the evidence of causality. Similarly, we found significantly higher risks in women than in men for BQ without tobacco studies in the Indian subcontinent. This suggests that women could be more susceptible than men to develop betel quid-induced oral cancer or could reflect different use patterns. It is probable that women may chew more quids per day. Buccal mucosa in our and the Guha et al paper was reported as the most common sub-site for oral cancer, the site where betel quid is usually placed and retained by the chewers. Furthermore, our meta-analysis has clearly separated the role of smokeless tobacco in all its forms from that of betel quid chewing without tobacco. It is clear that it is ST itself which is the major carcinogen in these communities.

### Limitations

Most studies did not use WHO ICD-10 codes, so the cancer sites associated with particular habits are imprecise [48], [53], [54], [57], [59], [64], [65]. Although tobacco chewing is common amongst women in India, Taiwan and Papua New Guinea, however, very few studies have reported information on oral cancer in women. Similarly, some studies [46], [66] reported consumption of chewing tobacco and/or of betel quid without tobacco as a multivariate variable (current, ever, ex-chewer, never). This inconsistent exposure assessment might have been further exacerbated by almost unavoidable recall bias in case-control studies. Studies conducted for betel quid without tobacco and incidence for oral cancer showed heterogeneity. Where possible, we explored this further using sensitivity analysis of the effects of excluding outlying studies [42]. Very few studies consistently evaluated graded doses and duration of consumption of ST which would have provided evidence of dose response relationship. Studies included in this meta-analysis varied in number and type of confounding variables and methods used to account for confounding. Therefore, residual confounding in the effect estimates cannot be ruled out. Uniformity in these methodological aspects would have provided un-biased effect estimates - an issue which needs to be addressed in future research.

### Conclusions

This meta-analysis clearly shows that ST NOS and BQ without tobacco are risk factors for oral cancers in Asia and the Pacific. For ST NOS there are sufficient studies which have adjusted for confounding by smoking and alcohol consumption, to show that the risk remains increased significantly across all intra-oral subsites: males are affected predominantly. Control of ST NOS and BQ without tobacco must remain an integral part of tobacco control in any public health strategy. Furthermore, governments around the world should prohibit the import of all ST products.

## Supporting Information

Table S1 **PRISMA checklist.**
(DOC)

Table S2 **Reason for excluding studies from meta-analysis.**
(DOCX)
