# Supplementary material for: Systematic Review and Meta-Analysis of Association of Smokeless Tobacco and of Betel Quid without Tobacco with Incidence of Oral Cancer in South Asia and the Pacific
Source: PLoS One. 2014 Nov 20;9(11):e113385. doi: 10.1371/journal.pone.0113385 (PMC4239077; doi:10.1371/journal.pone.0113385)
Supplement: Table S2 — Reason for excluding studies from meta-analysis. (DOCX) [file pone.0113385.s002.docx]

| **Supplementary Table 2. Criteria for excluding studies from meta-analysis** | | | | | | | |
| --- | --- | --- | --- | --- | --- | --- | --- |
| **s.no** | **Reference Type** | **Region** | **Time frame of study** | **Design of research study** | **Gender** | **Sample size** | **Reasons for exclusion** |
| 1 | Gupta PC,2005 | India | 1992-94 | Cohort | M+F | 99570 | Estimated mortality instead of incidence |
| 2 | Padnekar,2011 | India | 1991-97 | Cohort | M | 88,658 | Calculated Hazard's ratio instead of relative risk as an effect size of the study. Both relative risk and hazard's ratio are statistically different and cannot be assumed as same |
| 3 | Wen CP,2010 | Taiwan | 1994-2006 | Cohort | M | 177,271 | Mortality hazard ratios (HR) were estimated by Cox proportional hazard model to estimate mortality from oral cancer, hence excluded from analysis |
| 4 | Bile KM, 2010 | Pakistan | 2004-08 | Retrospective hospital based review | M+F | 21 245 | No control group to make comparison with data on cases |
| 5 | Sanghvi LD, 1995 | India |  | Retrospective hospital based review | M+F | 1460 | No control group to make comparison with data on cases |
| 6 | Wahi PN (1965 ) | India | 1950-62 | Case-control study | M+F |  | Adjusted OR and statistical analyses were not clearly stated or it was not possible to compute the effect estimate from the information given, Not able to separate oral from oropharyngeal cancer for statistical analysis |
| 7 | Chen PC,2011 | Taiwan | 1994-97 | Case-control study | M+F | 27 | Sample size of a case-control study was less than 50 |
